# Supplementary material for: Splice-Junction-Based Mapping of Alternative Isoforms in the Human Proteome
Source: Cell Rep. Author manuscript; Available in PMC 2020 Jan 15. (PMC6961840; doi:10.1016/j.celrep.2019.11.026)
Supplement: 2 [file NIHMS1546469-supplement-2.zip › DF1/PXD006675/RightAtrium/RightAtrium_12_UQCRH_MLTESGDPEEDPLTTVR.pdf]

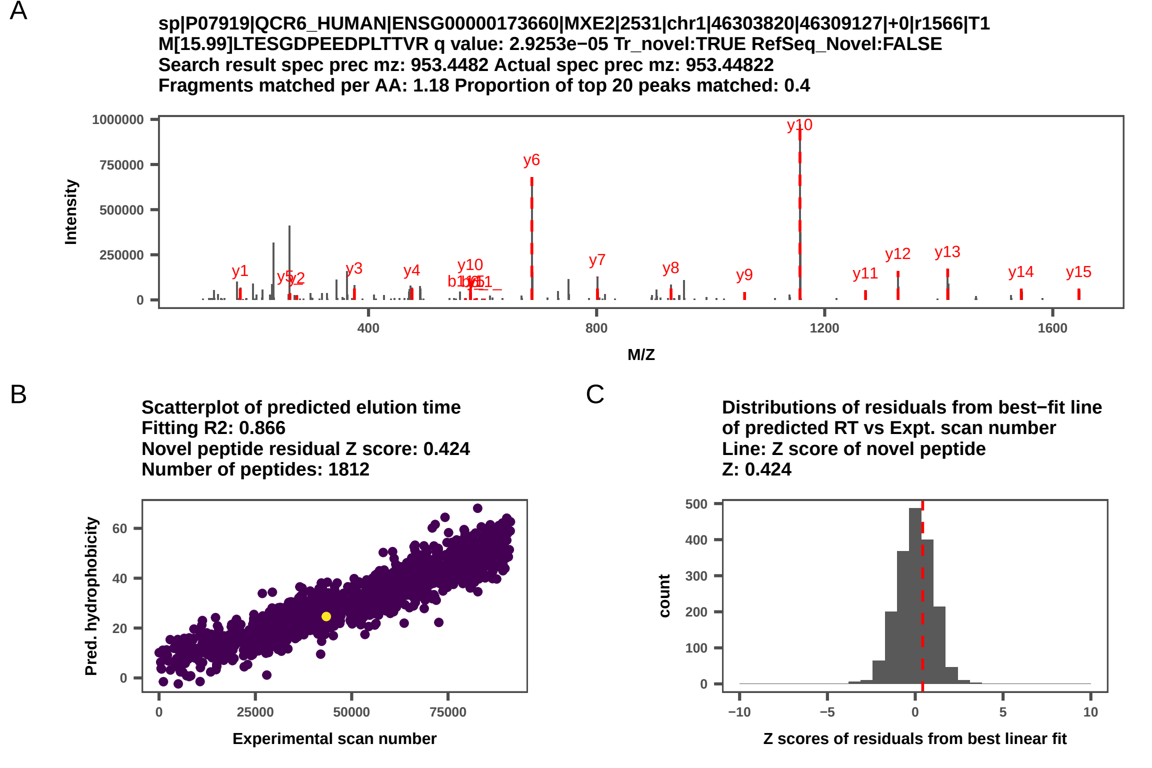
A
sp|P07919|QCR6_HUMAN|ENSG00000173660|MXE2|2531|chr1|46303820|46309127|+0|r1566|T1
M[15.99]LTESGDPEEDPLTTVR q value: 2.9253e−05 Tr_novel:TRUE RefSeq_Novel:FALSE
Search result spec prec mz: 953.4482 Actual spec prec mz: 953.44822
Fragments matched per AA: 1.18 Proportion of top 20 peaks matched: 0.4
Intensity
1000000
750000
500000
250000
0
y1
y5
y2
y3
y4
y10
y6
y7
y8
y9
y10
y11
y12
y13
y14
y15
400
800
1200
1600
M/Z
B
Scatterplot of predicted elution time
Fitting R2: 0.866
Novel peptide residual Z score: 0.424
Number of peptides: 1812
Pred. hydrophobicity
60
40
20
0
0
25000
50000
75000
Experimental scan number
C
Distributions of residuals from best−fit line
of predicted RT vs Expt. scan number
Line: Z score of novel peptide
Z: 0.424
count
500
400
300
200
100
0
−10
−5
0
5
10
Z scores of residuals from best linear fit
